# 

**Published:** 1889-05-11

**Authors:** 


### The Student's Column.

### EDINBURGH UNIVERSITY MEDICAL EXAMINATION.

The following is the official list of candidates who passed the second professional examination in medicine in April, 1889:

T. S. Adair, E. H. Alexander (with distinction), James Allan, J. D. C. Allen, W. S. Anderson, D. H. Balfour, W. F. Bauchop, J. H. Bayley, R. M. Beattie, W. H. H. Bennett, J. R. Bibby, Alfred Bienemann, W. A. Black, J. A. Bower, William Bower, W. H. Boazman, G. E. Bowker, P. H. Bowden, J. E. Brandt, T. M. Brown, F. L. Brown (M.A.), Robert Brown, S. B. J. Bulteel, J. R. Burnett, Percy Capper, William Carmichael, G. C. Cathcart, H. M. Chasseaud, R. D. Clarkson (with distinction), C. J. Van Coller, Robert Cran (with distinction), H. G. Critchley, B.A., S. B. Davis, A. J. Dearden, J. A. Dick, D. S. Dixson, W. B. Drummond (with distinction), Alexander Dunbar, A. S. Duncan, George Elder (with distinction), F. W. Eurich, O. F. Evans, R. M. Fenn, E. C. Fischer, George Fleming, Samuel Fleming, J. W. Forbes, J. A. Foulis, D. C. Gardiner, S. A. Gibbs, E. V. Gibson, H. J. Glover, E. H. Gonin, A. C. E. Gray, William Griffith, C. N. Hamper, J. M. Hermon, O. B. Hollard, G. J. B. Hope, R. M. Horne (with distinction), John Howard-Jones, James Huskie, W. M. Hutton, A. E. S. Jack, G. B. Jameson, J. H. Johnson, E. H. Jones, A. B. Kenworthy, W. S. Kerr (with distinction), J. G. Kershaw, J. J. J. Kotzé, J. R. P. Lambert, L. T. Lancaster, Thomas Lawson, James Lawson Williams, W. L. Legg, W. F. C. Lowson, J. D. L. Macalister, A. J. M'Callum, A. G. Macdonald, John Macdonald, T. F. Macdonald, Archibald Mackintosh, F. R. Mallett, R. B. Martin, J. Ackworth Menzies (with distinction), H. N. D. Milligan, T. H. Milroy, R. U. Moffat, J. R. Monnier, J. M'C. Morrison, C. D. Murray, J. S. M. Nurse, W. H. Ogilvie, W. H. B. O'Neill, John Orr (with distinction), T. W. Parkinson, W. J. Peddie, W. D. Rankin (B.A.), J. M. Reid, J. A. Robertson, W. I. Robertson, J. S. Y. Rogers, Murdo Rose (M.A.), H. G. F. C. le Roux, H. A. Rowland, J. L. Rubridge, Alfred Rutter, G. H. Rutter, J. G. V. Sapp (with distinction), Theodore Shennan, W. A. Simpson, J. F. Smith (M.A.), John Spurway, T. E. K. Stansfield, Thomas Steele, R. S. Stephenson, E. M. Steven, Frank Stephenson, Russell Sturgis-White, C. D. Sutherland, A. E. Taylor, J. E. Thomas, A. G. Thompson, Alexander Thomson, H. T. Thomson, J. E. Thomson, J. M. Thomson, George Thornton, Peter Thornton, Hubert Tibbits, F. R. Turton, Alexander Veitch, A. H. Walker, A. I. Webster, John Williams, J. D. Williams, S. L. N. Williams, G. M. Wilson, M. A. Wilson, J. M. Wood, David Young, D. P. Young (with distinction), J. B. Young, J. Y. S. Young.

**Notice to Advertisers and Subscribers.**

All Advertisements, Orders for Copies of The Hospital, and Business Communications should be addressed to The Publisher, *not to the Editor*, The Hospital, 139-140, Salisbury-court, Fleet-street, E.C.

Vol. V., now ready, 4s., post free. Cases for binding, may be had of the Publisher, at 1s. 6d. each.

**To Residents, Secretaries, and Matrons.**

The Editor will be glad to have the Regulations and each Report as issued by Asylums, Hospitals, Convalescent and Nursing Institutions, as well as those of other Charities, and an early copy in duplicate of all Appeals and Festival Notices, etc., addressed to *The Editor, The Lodge, Porchester-square, London, W.* Any superintendent, secretary, matron, or other chief official who regularly sends such information may be registered, on application to the Publisher, to receive free of cost a cover for binding The Hospital in half-yearly volumes.

### Amusements and Relaxation.

### Word Competition.

Commenced April 6th, Ends June 29th.

Three Prizes of 15s., 10s., 5s., will be given for the largest number of words derived from the words set for dissection.

Proper names, abbreviations, foreign words, words of less than four letters, and repetitions are barred; plurals, and past and present participles of verbs, are allowed. Nuttall's *Standard* dictionary only to be used.

N.B.—Word dissections must be sent in WEEKLY, arranged alphabetically, with correct total affixed.

The word for dissection for this, the *Sixth* week of the quarter being

"RITCHIE."

### Results of Fourth Week.

| Names. | May 2nd. | Totals. | Names. | May 2nd. | Totals. |
|---|---|---|---|---|---|
| Concours, Apl 25 | — | 126 | Eric | 16 | 310 |
| Patience | 18 | 387 | Tiny | 16 | 312 |
| Nurse Duty | 18 | 380 | N'importe Qui | 16 | 299 |
| Lightowlers | 17 | 376 | Pollie | 13 | 290 |
| Scrooge | — | 368 | W. G. R. | 13 | 270 |
| Reldas | 18 | 250 | Robe | — | 150 |
| Tinie | 18 | 390 | Beryl | 13 | 255 |
| K. G. | 18 | 362 | Nesta | — | 124 |
| Broxbourne | 18 | 368 | Daffodil | — | 89 |
| Lauriston | 17 | 368 | A Schoolgirl | 13 | 130 |
| Spes | 18 | 369 | Montague | — | 79 |
| Gretta | — | 224 | Sheeny | — | 111 |
| Francesca | 18 | 364 | Alice R. | — | 63 |
| Heatherbell | — | 185 | Belfast | — | 163 |
| Sycamore | — | 201 | Gentian | 17 | 209 |
| Arctus | 16 | 331 | Hope | — | 39 |
| Amicus | 16 | 355 | Louie | 11 | 139 |
| Scratcher | — | 197 | Hamilton | — | 121 |
| Jemima | — | 138 | E. M. | — | 120 |
| Concours | 11 | 314 | F. J. D. | — | 112 |
| C. J. S. | — | 134 | Ladyr | — | 102 |
| See-saw | 18 | 331 | Surgical Nurse | 16 | 115 |
| Rosemont | — | 173 | Violet | — | 95 |
| Jenny Wren | 16 | 142 | L. Pearce | — | 32 |

### Notice to all Correspondents.

N.B.—All letters referring to this page which do not arrive at 139 and 140, Salisbury-court, London, E.C., by the *first post on Thursdays*, and are not addressed PRIZE EDITOR, will in futu be disqualified and disregarded.

### Conditions.

I. Every paper sent in must be clearly written, and one side only must be used All competitors must mark at the head of their papers the number of their competition.

II. Each paper must be signed by the author with his or her real name and address A *nom de plume* may be added if the writer does not desire to be referred to by us by his real name. In the case of all prize-winners, however, the real name and address will be published.

III. All communications relating to prize competitions should be addressed to "The Prize Editor, The Hospital, 139 and 140, Salisbury-court, London, E.C." Competitors are requested not to include in letters, etc., to the Prize Editor communications on matters of other business. All letters must be fully prepaid.

IV. The Prize Editor of The Hospital is the sole judge of the competitions, and his decision is in all cases final and without appeal.

V. The Prize Editor reserves the right to publish any paper sent in, whether it receives a prize or not. He does not hold himself responsible for any MSS. sent, nor can he undertake to return rejected competitions.

### Scraps and Gleanings.

Miss Fellows has given a concert in aid of the Grimsby Hospital.

An epidemic of malignant scarlet fever is reported from New York.

Hereford General Infirmary has been able to invest £2,000 in the county rates.

The students of the London Hospital are going to give a concert on the 23rd of this month.

A successful ball was held last week at Eastbourne in aid of the Princess Alice Hospital.

The Chairman of the Hull Royal Infirmary has issued a special appeal for further subscriptions.

The Meistersingers Club gave an excellent May Day medley in aid of the Hospitals Association.

During Whit Week a bazaar will be held at Stanley Park in aid of the Stanley Hospital, Liverpool.

The Princess of Wales has sent a contribution to the bazaar in aid of the Bootle Borough Hospital.

Last Saturday was the ninth anniversary of Hospital Saturday at Bolton. Collections were made.

Dr. Mears has resigned his post as lecturer on anatomy to the College of Medicine, Newcastle-on-Tyne.

Mr. Herbert Lund, F.R.C.S., has been appointed honorary assistant surgeon to the Salford Royal Hospital.

The profits of the Private Nursing Institution attached to Grimsby Hospital amounted to about £120 per annum.

During April a fire occurred at the Royal Free Hospital, but was promptly extinguished by a courageous nurse.

In Manchester for the week ending April 27, the birth-rate was equal to 36·6 per thousand, and the death-rate to 28·4.

A successful concert was held at Aberdeen on April 29th, in aid of the Children's Hospital. Lord Saltoun presided.

One thousand medical, and two thousand surgical, cases were attended to at the Great Northern Hospital during April.

On May 29th a meeting will be held at Dudley House in aid of the Hospital for Sick Children, Great Ormond-street.

Sir Henry Thompson, Mr. Ernest Hart, and Mr. Critchett were all present at the private view of the Royal Academy.

St. Mary's Cottage Hospital, Tenbury, report says that 69 patients were treated last year, at an average cost of 7s. per week per head.

The Church of England Temperance Society held its 27th anniversary last Saturday, in Westminster Town Hall. Good progress was reported.

The *National Review* contains an article on Doctoring in China, and *Longmans Magazine* contains an article on Father Damien among the Lepers.

The last lecture of the winter course at the Sanitary Institute takes place on May 16th, when Mr. Davis will speak on "Fires and Fire Escapes."

Sir James Sawyer has resigned his post of physician to the Queen's Hospital, Birmingham. Sir James has been connected with the hospital since 1861.

Sir John Aird is doing his duty as chairman of St. Mary's Hospital festival dinner. He has issued a pressing appeal for support and for contributions.

Elizabeth Snelson, a lunatic pauper, who recently died in Macclesfield Lunatic Asylum, cost the Altrincham Union over £900 since her incarceration in 1847.

Queen Margherita of Italy is very charitable; but the form of charity in which she most delights is playing the 'cello at concerts given for charitable purposes.

The Emperor of Germany has conferred the Red Eagle, Third Class, on Dr. Felix Semon, assistant physician in charge of the Throat Department, St. Thomas's Hospital.

In connection with the forthcoming Exhibition at Paris an international congress on the housing of the poor will be held at Paris on the 26th, 27th, and 28th of June next.

Nine residents of Ashford (Kent) were last week summoned for neglecting to have their children vaccinated. Seven of them were fined 20s. and costs, and the other two 10s. and costs.

There is one lunatic asylum in America where the female patients are managed exclusively by women. It is that at Norristown, Pennsylvania, where Dr. Alice Bennett has charge over 800 women.

Professor Mendeleef, the famous Russian chemist, will deliver the triennial Faraday lecture at the Royal Institution, on June 4th, under the auspices of the Chemical Society of Great Britain.

A lady in her 103rd year, the late Miss Rebecca Norton, of Brentford, was buried on May 3, at Woking Cemetery, Brookwood, Surrey. The burial was conducted on the "earth-to-earth" system.

The Right Hon. Viscount Gough presided at the annual meeting of the Adelaide Hospital, Dublin, on April 25th. The report was satisfactory, except with regard to funds. More subscribers are urgently needed.

Mr. W. C. Bailey, of Edinburgh, who spent twelve years as a missionary amongst the lepers of Southern India, lectured lately at Halifax on the deficient hospital accommodation for such cases in some parts of the East.

The 1,463 deaths which occurred in London last week, included 65 from measles, nine from scarlet fever, 29 from diphtheria, 56 from whooping-cough, three from enteric fever, 18 from diarrhœa and dysentery, and not one from small-pox, typhus, or cholera.

Last week there were no fewer than 2,806 bathers in the baths of Bath, and the receipts from bathers and water-drinkers amounted to £200. During the last five weeks the receipts at the various baths have been £130 in excess of the amount during the corresponding weeks of last year.

